# Association Between Conservative Treatment Modalities and Clinical Outcomes in Patients With Lumbosacral Disc Herniation Following Short Term and Long Term Treatments: A Retrospective Cohort Study

**DOI:** 10.1002/hsr2.73085

**Published:** 2026-08-26

**Authors:** Bina Eftekharsadat, Shadi Khodaei, Raha Markazi‐Movaghar, Fatemeh ojaghlou, Azizeh Farshbaf‐Khalili, Soraya Babaie

**Affiliations:** ^1^ Physical Medicine and Rehabilitation Research Center, Aging Research Institute Tabriz University of Medical Sciences Tabriz Iran; ^2^ Faculty of Medicine Tabriz University of Medical Sciences Tabriz Iran

**Keywords:** Back pain, conservative treatment, intervertebral disc displacement, surgery

## Abstract

**Background and Aims:**

Lumbar disc herniation (LDH), a leading cause of low back pain, necessitates effective conservative management to mitigate surgical interventions. This retrospective observational study evaluated the short‐term (6 months–2 years) and long‐term (3–6 years) efficacy of conservative therapies in 194 LDH patients from Iran.

**Methods:**

Data were collected via medical records, demographic questionnaires, and the numerical rating scale (NRS) for pain, with analyses adjusting for covariates such as body mass index (BMI), comorbidities, and treatment modalities. Data were analyzed using SPSS 24 (Kolmogorov–Smirnov normality test), including descriptive (frequencies, means/SDs) and inferential statistics (*t*‐tests, ANOVA, chi‐square, Fisher's exact); ANCOVA and multivariate logistic regression (adjusted for confounders, 95% confidence interval CI) modeled pain/paresthesia outcomes.

**Results:**

The **s**ignificant pain reduction after treatment (mean NRS: 7.3 to 3.7, *p* < 0.001) was demonstrated with home exercise programs showing the largest improvement (adjusted *β*: −0.74, *p* = 0.010). Short‐term pharmacological therapy (≤ 24 months) also reduced pain (*β* = −0.96, *p* = 0.02), while long‐term use lacked efficacy. Higher BMI correlated with higher pain (*p* = 0.03) and paresthesia risk (*p* = 0.01), and water therapy unexpectedly increased paresthesia odds (odds ratio (OR) = 4.14, *p* = 0.03) compared to other treatments. Females reported higher paresthesia rates (52.7% vs. 33.3%, *p* = 0.03), and comorbidities like hypertension were associated with a higher rate of paresthesia. Satisfaction rates exceeded 66% across therapies, though adherence to acupuncture, yoga, and injections was low.

**Conclusion:**

The findings suggest that home‐based exercise and short‐term pharmacological therapy were associated with more favorable pain outcomes in this cohort. However, due to the observational design, these findings should be interpreted as associations rather than evidence of causal treatment effectiveness.

**Abbreviations**
BMI: body mass index
CI: confidence interval
LBP: low back pain
LDH: lumbar disc herniation
MRI: magnetic resonance imaging
NRS: numerical rating scale
OR: odds ratio
PNE: Pain Neuroscience Education

## 1 Introduction

Low back pain affects nearly 80% of individuals at least once in their lifetime, with lumbar disc herniation (LDH) representing a leading etiology. LDH most frequently involves the L4–L5 and L5–S1 spinal segments and is primarily driven by intervertebral disc degeneration [1, 2]. Globally, LDH exhibits an average prevalence of 14%–20% and an incidence rate of 2%–4%, with the majority of cases occurring among adults aged 30–50 years [3, 4]. Diagnosis involves a thorough history, physical examination, and imaging [5].

LDH is associated with several pathophysiological changes in the intervertebral disc, including reduced water retention in the nucleus pulposus [6], increased type I collagen in the nucleus and inner annulus fibrosus, degradation of collagen and extracellular matrix components [7], and activation of pathways such as apoptosis, matrix metalloproteinase expression, and inflammatory signaling [7]. Genetic predisposition plays a significant role, with heritability estimated at 75% [8]. Key susceptibility genes encode structural proteins, matrix metalloproteinases, apoptosis regulators, growth factors, and vitamin D receptor polymorphisms, which drive inflammatory cytokine imbalances [7].

Clinical manifestations of LDH include radicular pain, sensory abnormalities, and weakness in lumbar–sacral nerve root distributions. Pain exacerbation with trunk flexion, Valsalva maneuvers (e.g., coughing), and prolonged sitting, which increases disc pressure by 40%, is characteristic [9].

LDH treatment can be conservative or surgical, with ongoing debate about their relative effectiveness. Surgical intervention provides faster relief from back pain symptoms in the short term compared to conservative therapy [10]. While discectomy provides rapid leg pain relief and satisfaction in 65–90% of cases, and injections yield short‐term benefits for 50–85% of patients, these interventions entail risks and high costs. However, long‐term outcomes show minimal differences between the two approaches [11]. Surgery may be beneficial for patients with debilitating pain seeking rapid relief or those who haven't improved with conservative treatment [11]. In most cases, treatment typically begins with conservative approaches and remains the first‐line strategy for most patients. This approach generally includes activity modification, pharmacological therapy, physical therapy, and, in selected settings, complementary and integrative interventions [12, 13, 14]. Studies have shown that conservative treatment can be effective, with 51% of patients avoiding surgery and experiencing long‐term improvements in pain and neurological symptoms [15]. If conservative methods fail or major neurological symptoms persisted over 6 weeks, surgery may be recommended [16]. Operative treatment, particularly minimally invasive endoscopic microdiscectomy, is currently the gold standard for severe cases, showing the best results for postoperative pain and function [17]. However, no single treatment method has been definitively proven superior [18]. Moreover, despite the efficacy of initial conservative strategies, the long‐term utility of these therapies remains unclear. Selecting optimal conservative treatments balancing efficacy, safety, cost, and accessibility poses a persistent challenge. Prior studies report conflicting evidence on intermediate‐ to long‐term outcomes, underscoring the need for rigorous evaluation. This study aims to assess the effectiveness of conservative therapies following short‐term and long‐term treatments in patients with LDH to identify factors influencing treatment quality and reduce unnecessary spinal surgeries.

## 2 Material and Methods

### 2.1 Study Design and Setting

This was a multicenter retrospective observational cohort study conducted using electronic medical records of patients with LDH who received conservative treatment at four physical medicine and rehabilitation clinics affiliated with Tabriz University of Medical Sciences. This study was conducted and reported in accordance with the Strengthening the Reporting of Observational studies in Epidemiology (STROBE) statement [19]. It was conducted following approval by the Ethics Committee of Tabriz University of Medical Sciences on April 10, 2025 (approval code: IR.TBZMED.REC.1403.1112) and authorization from the Vice Chancellor of Research. All activities carried out in research, including human subjects, were in accordance with the ethical principles established by the institutional and/or national research board, as well as the 1964 Helsinki declaration and subsequent revisions, or similar ethical guidelines. The study population comprised all adults with LDH who were not candidates for surgical intervention and had electronic records at educational‐therapeutic hospitals in Tabriz.

Among the ten tertiary care hospitals in the city, five institutions maintain specialized physical medicine and rehabilitation clinics. A computerized randomization protocol was implemented through the Random.org platform, selecting four clinical sites excluding the pediatric rehabilitation center (Pediatrics MardaniAzar Hospital). A proportional random sampling method was used to select the required number of participants (48 or 49 per center) from a list of eligible patients with electronic records at the mentioned centers, with all data subsequently anonymized and provided to researchers in coded format. This sampling framework optimized demographic proportionality while maintaining methodological rigor in participant selection.

Electronic medical records of patients treated between 2018 and 2024 were reviewed during the data collection period (December 2023–September 2024). Outcomes were extracted according to the elapsed time since initiation of conservative treatment, allowing classification into short‐term (6 months–2 years) and long‐term (3–6 years) follow‐up groups. The authors had access to information that could identify individual participants during or after data collection to facilitate phone calls and gather essential information. The primary objective of this study was to evaluate the effectiveness of conservative treatment modalities in reducing pain intensity, as measured by the numeric rating scale (NRS), among patients with LDH during short‐term and long‐term follow‐up. The secondary objective was to assess the association between conservative treatment modalities and paresthesia outcomes over the same follow‐up periods.

### 2.2 Participants

The study participants were recruited from physical medicine and rehabilitation clinics affiliated with educational‐medical institutions in Tabriz, Iran, following established inclusion/exclusion parameters. Inclusion Criteria consisted of: (1) Confirmed diagnosis of LDH via magnetic resonance imaging (MRI) with no surgical indications. (2) Provision of oral informed consent for study participation. Exclusion Criteria comprised: (1) Progressive neurological deficits, (2) Urinary or fecal incontinence, (3) Grade 3 or 4 spondylolisthesis, or dynamic grades 1–2, (4) Concurrent cervical myelopathy, (5) Diabetic neuropathy or other neuropathic disorders, (6) Prior receipt of conservative therapies for LDH with reported dissatisfaction, (7) Documented psychiatric or comorbid medical conditions (e.g., inflammatory diseases, trauma, malignancies), (8) History of spinal surgery.

### 2.3 Sample Size

The sample size was calculated based on the mean pain intensity reported for patients receiving long‐term conservative treatment for LDH in the study by Peul et al. [20]. Using the single‐population mean formula (*n* = Z^2^S^2^/d^2^), with a standard deviation (S) of 27.7, a 95% confidence level (*Z* = 1.96), and an absolute precision (margin of error) of 4.5 units, the minimum required sample size was estimated to be 142 participants. To account for an anticipated 20% attrition rate, the target sample size was increased to 170 participants.

### 2.4 Sampling Procedure

Among the ten tertiary hospitals in Tabriz, five maintain physical medicine and rehabilitation clinics. Four clinics were randomly selected using Random.org after excluding the pediatric rehabilitation center. Eligible patients with MRI‐confirmed LDH were identified from electronic medical records. Proportional random sampling was then used to recruit approximately 48–49 participants from each center until the required sample size (*n* = 170) was achieved. The investigation initiated with structured consultations at multidisciplinary physical medicine and rehabilitation centers, where researchers articulated the study's scientific aims to participating clinicians. Clinical archives were conducted to isolate LDH‐diagnosed patients undergoing non‐operative management protocols. Subsequently, a comprehensive analysis of therapeutic documentation was performed to codify clinician‐prescribed conservative modalities, with particular attention to intervention typologies, temporal patterns of therapeutic application, and longitudinal treatment algorithms. A retrospective analysis was conducted to evaluate short‐term (6 months–2 years) and long‐term (3–6 years) outcomes of conservative treatments.

Clinical data were extracted from eligible participants' electronic medical records using a standardized data extraction form. Information obtained from medical records included baseline clinical characteristics, MRI‐confirmed level and severity of LDH, pre‐treatment pain intensity, disease duration, prescribed conservative treatment modalities, treatment adherence, comorbidities, and other relevant clinical findings. Subsequently, eligible patients were contacted by telephone to obtain verbal informed consent and complete the study questionnaires. Telephone interviews were used to collect demographic information not available in the medical records, current pain intensity using the NRS, the presence or absence of paresthesia, patient satisfaction with conservative treatment, subsequent surgical referral, and other follow‐up outcome data. Prior to questionnaire administration, verbal informed consent was obtained from all participating patients, who also consented to the use of their medical record data for research purposes. Conservative treatment modalities documented in the medical records included pharmacological therapy, physiotherapy, home exercise programs, acupuncture, therapeutic injections, hydrotherapy (water therapy), massage therapy, and yoga. Treatment protocols reflected routine clinical practice. Pharmacological treatments included medications prescribed for symptom control according to clinical judgment. Physiotherapy consisted of individualized rehabilitation programs including modalities and therapeutic exercises. Home exercise programs were prescribed according to patients' clinical conditions and functional status. Injection techniques and hydrotherapy protocols were performed according to clinician preference; however, detailed procedural parameters were not consistently available in retrospective records. Because conservative care was delivered according to routine clinical practice, some patients received more than one treatment modality during the follow‐up period. Treatment modalities were therefore considered as independent exposures rather than mutually exclusive treatment groups. For each participant, the type of conservative treatment received and its duration were recorded. Subsequent surgical intervention during the follow‐up period was also documented as a clinical outcome rather than a conservative treatment modality.

### 2.5 Data Collection

This study utilized structured questionnaires for data collection, comprising the following components:

1. Eligibility checklist: To screen and enroll participants meeting predefined inclusion criteria.
2. Demographic questionnaire: Captured variables including age, gender, marital status, educational attainment, employment status, income, body mass index (BMI), ethnicity, comorbidities, presence or absence of paresthesia, and severity/level of disc herniation. This questionnaire was developed based on study objectives and a review of scientific literature. The validity of this questionnaire was determined by eight academic members as a panel of experts.
3. NRS: Assessed pain intensity.
4. Intervention satisfaction checklist: Evaluated patient satisfaction with received treatments.

The NRS is a validated, patient‐friendly tool for quantifying pain intensity. Participants rated their pain on an 11‐point scale (0–10), where 0 indicates “no pain,” and 10 represents “worst imaginable pain.” This scale is widely used due to its simplicity, strong correlation with the Visual Analogue Scale (VAS) [21], and adaptability for verbal administration without requiring visual or motor coordination [22]. Its preference over VAS in clinical settings stems from enhanced ease of comprehension and application [22, 23].

### 2.6 Statistical Analysis

Statistical analyses were performed using IBM SPSS Statistics for Windows, version 24.0 (IBM Corp., Armonk, NY, USA). Normality of distribution was assessed via the Kolmogorov–Smirnov test. Analyses included: Descriptive statistics: Frequency distributions, means and standard deviations, and Inferential statistics: Independent *t*‐tests, one‐way ANOVA, chi‐square, trend chi‐square, and Fisher's exact test. To determine the association between post‐treatment pain intensity and paresthesia persistence after short‐ and long‐term conservative therapies, while controlling for confounding variables (e.g., concurrent treatments), the following models were employed: General linear model adjusted for covariates influencing pain outcomes. Multicollinearity among treatment‐related predictors included in the regression models was assessed using the variance inflation factor (VIF). A VIF value < 5 was considered indicative of absence of substantial multicollinearity. Multivariate logistic regression: Evaluated associations between treatment modalities and binary outcome (paresthesia presence/absence) adjusted for confounding variables (e.g., concurrent treatments), with a 95% confidence interval. Model calibration was assessed using the Hosmer–Lemeshow goodness‐of‐fit test. Variables with *p* < 0.20 in univariate analyses and clinically relevant covariates were entered into multivariable models. The primary analyses comparing pre‐ and post‐treatment pain intensity and paresthesia as well as subgroup analyses according to treatment duration and treatment modality were pre‐specified. Statistical significance was defined a priori as a two‐sided *p* < 0.05. Statistical analyses and reporting were performed according to the recommendations of Assel et al. for reporting statistics in clinical research [24] and the Statistical Analyses Methods in the Published Literature (SAMPL) reporting guidelines [25].

## 3 Results

**Figure 1:**
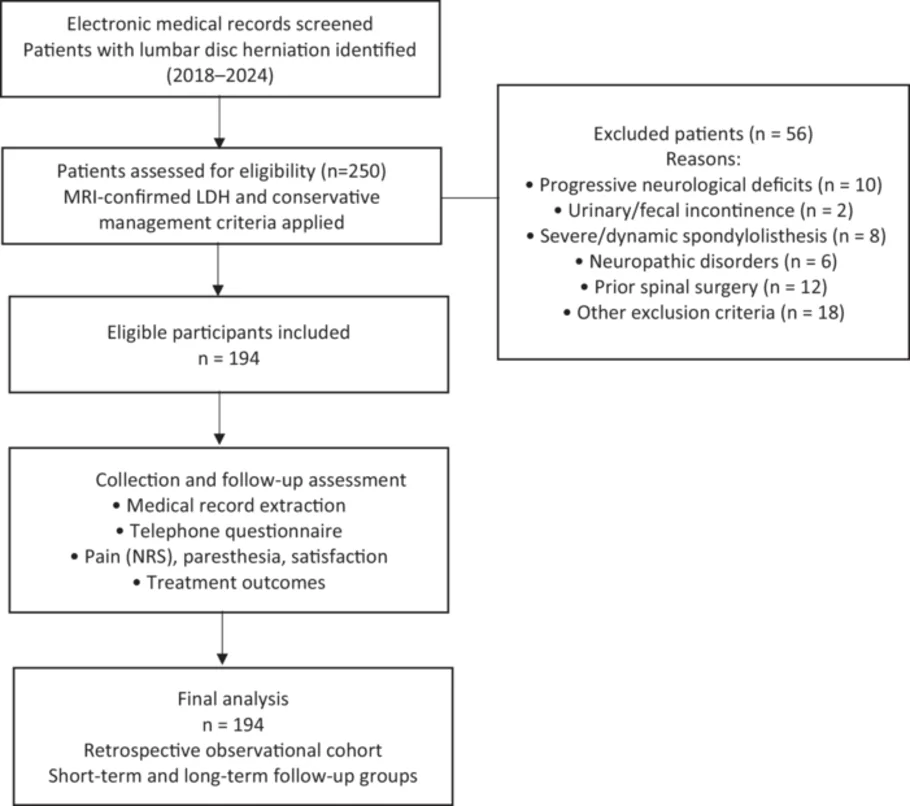
STROBE flow diagram of participant selection and analysis.

This retrospective observational cohort study included 194 patients with LDH who received conservative treatment. The methodological framework comprised three sequential phases: (1) comprehensive electronic health record screening using standardized diagnostic codes, (2) application of predefined exclusion parameters to optimize cohort homogeneity, and (3) implementation of structured telephonic engagement procedures. A total of 194 patients with LDH who received conservative treatment met the eligibility criteria and were included in the final analysis (Figure 1).

The mean (SD) age and BMI of participants were 55.4 (11.9) years and 25.6 (2.8) kg/m^2^, respectively. Of the 194 participants, 75.3% were women, 63.1% were overweight, and 62.2% had at least one comorbidity. More than 40% of study participants had a diploma and two‐thirds of them were housewives. The detailed demographic and clinical characteristics were shown in Table 1 and Appendix 1.

**Table 1:** The demographic characteristics of patients with lumbar disc hernia and their relation with pain intensity and paresthesia (194 people).

Variable	*n* (%)	NRS (0–10) Mean (SD)	*p* value	Paresthesia *n* (%)	
^†^Age (years)/Mean ± SD	55.4 (11.9)	7.3 (1.0)	*r* = 0.01, *p* = 0.895	93 (47.7%)	
22–40	24 (12.4)	7.5 (1.1)	*p* = 0.154* df = 4, *F* = 1.6	9 (9.7%)	*p* = 0.637^¥^ df = 4 Chi^2^ = 2.5
41–50	37 (19.1)	6.9 (1.2)		18 (19.4%)	
51–60	61 (31.4)	7.4 (0.9)		26 (28%)	
61–70	51 (26.3)	7.4 (0.9)		29 (31.2%)	
71–83	21 (10.8)	7.2 (0.9)		11 (11.8%)	
^†^BMI (kg/m^2^)/Mean ± SD	25.6 (2.8)	7.3 (1.0)	***p*** = 0.034* df = 2, *F* = 3.4		***p*** = **0.012** ^¥^ df = 2 Chi^2^ = 8.9
Normal	49 (26.2)	7.0 (0.9)		16 (18.4%)	
Overweight	118 (63.1)	7.4 (1.1)		57 (65.5%)	
Obese	20 (10.7)	7.7 (0.9)		14 (16.1%)	
Gender			*p* = 0.076^±^ df = 1, *F* = 3.2		0.030^£^
Female	146 (75.3)	7.4 (1.0)		77 (82.8%)	
Male	48 (24.7)	7.1 (1.0)		16 (17.2%)	
Marital status			*p* = 0.813^±^ df = 1, *F* = 0.81		*p* = 1.0^£^
Single	2 (1.1)	7.5 (2.1)		1 (1.1%)	
Married	189 (98.9)	7.3 (1.0)		91 (98.9%)	
Occupation			*p* = 0.107* df = 3, *F* = 2.1		*p* = 0.078^¥^
Housewife	130 (69.5)	7.4 (1.0)		69 (77.5%)	df = 3
Part time occupied	30 (16.1)	7.1 (1.1)		8 (9%)	Chi^2^ = 6.8
Full time occupied	6 (3.2)	8.2 (0.7)		3 (3.4%)	
Retired	21 (11.2)	7.2 (0.8)		9 (10.1%)	
Smoking			*p* = 0.350^±^ df = 1, *F* = 0.87		*p* = 0.339^£^
Smoker	10 (5.3)	7.6 (0.84)		3 (3.3%)	
Non‐smoker	180 (94.7)	7.3 (1.0)		87 (96.7%)	
Household income			*p* = 0.166^±^ df = 1, *F* = 1.9		0.695^§^ df = 1 Chi^2^ = 154
Adequate	122 (70.9)	7.2 (1.1)		55 (69.6%)	
Rather adequate	50 (29.1)	7.4 (0.9)		24 (30.4%)	
Habitation					0.582^£^
Urban	180 (92.8)	7.3 (1.0)	*p* = 0.476^±^ df = 1, *F* = 0.51	85 (91.4%)	
Rural	14 (7.2)	7.1 (1.2)		8 (8.6%)	
Education					*p* = 0.150^§^
Under diploma	38 (22.6)	6.9 (0.7)	***p*** = 0.026* df = 2, *F* = 0.51	21 (26.3%)	df = 1
Diploma	70 (41.7)	7.5 (1.1)		35 (43.8%)	Chi^2^ = 2.1
Academic	60 (35.7)	7.5 (1)		24 (30%)	
Other diseases			*p* = 0.784^±^ df = 1, *F* = 3.7		***p*** = **0.003** ^£^
No	73 (37.8)	7.3 (1.2)		24 (25.8%)	
Yes	120 (62.2)	7.3 (0.9)		69 (74.2%)	
Depression			*p* = 0.084 ^±^ df = 1, *F* = 3.0		*p* = 1.0^£^
No	191 (98.5)	7.3 (1.0)		92 (98.9%)	
Yes	3 (1.5)	8.3 (1.1)		1 (1.1%)	
Diabetes without neuropathy			*p* = 0.373^±^ df = 1, *F* = 0.79		*p* = 1.0^£^
No	173 (89.2)	7.3 (1.0)		83 (89.2%)	
Yes	21 (10.8)	7.1 (0.8)		10 (10.8%)	
Knee problem			*p* = 0.272 ^±^ df = 1, *F* = 1.21		*p* = 1.0^£^
No	178 (91.8)	7.3 (1.0)		85 (91.4%)	
Yes	16 (8.2)	7.1 (0.7)		8 (8.6%)	
Rheumatism			*p* = 0.062^±^ df = 1, *F* = 3.5		*p* = 1.0^£^
No	192 (99)	7.3 (1.0)		92 (98.9%)	
Yes	2 (2)	6.0 (0)		1 (1.1%)	
Hypertension			*p* = 0.121 df = 1, *F* = 2.4		***p*** = **0.038** ^£^
No	123 (63.4)	7.2 (1.1)		52 (55.9%)	
Yes	71 (26.6)	7.5 (0.9)		41 (44.1%)	
Gastrointestinal disease			*p* = 0.344 df = 1, *F* = 0.90		***p*** = **0.045** ^£^
No	172 (88.7)	7.4 (1.0)		78 (83.9%)	
Yes	22 (11.3)	7.1 (0.7)		15 (16.1%)	
Cardiovascular disease			*p* = 0.196^±^ df = 1, *F* = 1.68		*p* = 0.155^£^
No	186 (95.9)	7.3 (1.0)		87 (93.5%)	
Yes	8 (4.1)	6.9 (0.6)		6 (6.5%)	
Thyroid disease			*p* = 0.990^±^ df = 1, *F* = 0.01		*p* = 0.582^£^
No	182 (93.8)	7.3 (0.9)		85 (91.4%)	
Yes	12 (6.2)	7.3 (1.3)		8 (8.6%)	
Hernia type			*p* = 0.287* df = 1, *F *= 1.25		*p* = 0.642^¥^
Protruding	103 (54.8)	7.4 (1.1)		49 (55.7%)	df = 2 Chi^2^ = 0.887
Listhesis**	66 (35.1)	7.3 (0.9)		29 (33%)	
Bulging	19 (10.1)	7.0 (0.9)		10 (11.4%)	
Hernia level			*p* = 0.452* df = 3, *F* = 0.88		*p* = 0.162^¥^ df = 3 Chi^2^ = 6.5
L2–L3, L3–L4	24 (12.7)	7.4 (1.3)		10 (11.1%)	
L4–L5	61 (32.3)	7.4 (0.9)		34 (37.8%)	
L5–S1	37 (19.6)	7.5 (1.1)		12 (13.3%)	
Multiple	67 (35.4)	7.2 (0.9)		34 (37.8%)	

*Note:* All numbers are reported as counts (percentages) except those marked with †, which represent the mean (standard deviation); *One‐way ANOVA, ^±^Independent *t*‐test; ^¥^ Chi‐square test; ^£^ Fisher's exact test; ^§^ Chi‐square by trend; NRS: Numeric Rating Scale for pain (0–10). Patients with knee problems had no need for surgery. Rheumatism patients were included if they were in a controlled state. ^**^Listhesis patients in non‐dynamic Grade I & II were included.

Significant associations were observed between demographic and clinical characteristics with and pain intensity. Higher BMI categories (overweight/obese) correlated with increased pain intensity (mean difference: 0.4–0.7, *p* = 0.03) and higher paresthesia prevalence (70%/48.3% vs. 32.6% in normal BMI, *p* = 0.01). Females exhibited significantly greater paresthesia rates (52.7% vs. 33.3%, absolute difference, 19.4%; *p* = 0.03), though pain intensity showed no gender‐based difference (*p* = 0.08). Comorbidities, particularly hypertension (57.7% vs. 42.3%, *p* = 0.04) and gastrointestinal diseases (68.2% vs. 45.3%, *p* = 0.04), were strongly linked to paresthesia. Participants with diploma or academic education had higher mean pain scores than those with lower educational attainment (7.5 vs. 6.9; mean difference, 0.6 points; *p* = 0.03). Age, hernia type/level, marital status, and smoking showed no significant associations (Table 1).

This pre‐post analysis of 194 LDH patients was associated with significant reductions in pain and paresthesia following treatment. Pain intensity, assessed via the NRS, decreased significantly 7.3 (SD: 1.0) to 3.7 (SD: 1.7), with a mean paired difference of −3.6 points (95% confidence interval (CI): −3.9 to −3.3, *p* < 0.001). Paresthesia frequency also improved significantly: patients reporting “no” symptoms increased from 52.3% to 76.4%, while those with “sometimes” or “constant” paresthesia declined from 42.6% to 20.0% and 5.1% to 3.6%, respectively (*p* < 0.001) (Table 2).

**Table 2:** The pain and paresthesia complications of patients with LDH before and after different treatments (194 people).

Variable	Baseline		After treatment		MD 95% CI	*p* value*
	Mean (SD)	**Min, Max**	Mean (SD)	**Min, Max**		
NRS (0–10)	7.3 (1.0)	3, 10	3.7 (1.7)	0, 10	−3.6 (−3.9 to −3.3)	< 0.001
Paresthesia	*n*	%	*n*	%		
No	102	52.3	149	76.4		< 0.001
Sometimes	83	42.6	39	20		
Constantly	10	5.1	7	3.6		

Abbreviations: CI, Confidence interval; MD, Mean difference; NRS, Numeric Rating Scale for pain (0–10).

* Paired sample *t*‐test.

Table 3 revealed differential pain reduction across treatment modalities. All interventions demonstrated significant intragroup improvements in pain intensity (NRS reduction *p* < 0.001). Participants performing home exercise experienced greater reductions in pain intensity than the other treatment modalities, with adherent patients reporting higher baseline pain (7.5 vs. 7.1, recipients vs. non‐recipients of home exercise), *p* = 0.01) but greater reduction in pain intensity after treatment (3.4 vs. 4.1, mean difference: −0.7 points; *p* = 0.004). Short‐term pharmacological therapy (≤ 24 months) showed lower post‐treatment pain intensity compared to non‐recipients (3.7 vs. 4.5, *p* = 0.01), while long‐term pharmacological therapy (3–6 years) showed no intergroup difference (*p* = 0.07). Physiotherapy recipients had higher baseline pain in short‐term cohorts (7.5 vs. 6.8, *p* = 0.002), though post‐treatment scores did not differ significantly (*p* = 0.21). Low‐adoption therapies (acupuncture [3.7%], surgery [1.6%], yoga [1%]) showed no significant intergroup effects (*p* > 0.05).

**Table 3:** The relation of different treatments with pain intensity among LDH people (194 people).

Variable	*n* (%)	NRS baseline (0–10)	*p* value Intergroup	NRS after treatment (0–10)	*p* value Intergroup	*p* value Intragroup
Pharmacological therapy						
Yes	83 (43.2)	7.4 (0.9)	3.7 (1.4)	< 0.001
No	109 (56.8)	7.3 (1.0)	3.7 (1.8)	< 0.001
Short treatment			0.126		**0.015**	
Yes	47 (48.4)	7.4 (0.9)		3.7 (1.3)		
No	50 (51.6)	7.1 (1.1)		4.5 (1.8)		
Long treatment			0.827		0.067	
Yes	36 (37.9)	7.4 (0.9)		3.6 (1.7)		
No	59 (42.1)	7.4 (1.0)		3.0 (1.4)		
Physiotherapy			0.075		0.307	
Yes	113 (58.9)	7.4 (0.9)		3.6 (1.6)		< 0.001
No	79 (41.1)	7.2 (1.1)		3.8 (1.6)		< 0.001
Short treatment			**0.002**		0.214	
Yes	65 (67.0)	7.5 (0.8)		3.9 (1.6)		
No	32 (33.0)	6.8 (1.2)		4.4 (1.7)		
Long treatment			0.725		0.260	
Yes	48 (67)	7.4 (0.9)		3.1 (1.6)		
No	47 (33)	7.4 (1.0)		3.4 (1.5)		
Home exercise			**0.011**		**0.004**	
Yes	105 (55)	7.5 (1.1)		3.4 (1.5)		< 0.001
No	86 (5)	7.1 (0.9)		4.1 (1.7)		< 0.001
Short treatment			**0.013**		0.062	
Yes	41 (42.3)	7.5 (1.2)		3.7 (1.7)		
No	56 (57.7)	7.1 (0.9)		4.4 (1.6)		
Long treatment			0.410		0.319	
Yes	65 (68.4)	7.4 (0.9)		3.1 (1.4)		
No	30 (31.6)	7.3 (0.9)		3.5 (1.8)		
Acupuncture			0.167		0.522	
Yes	7 (3.7)	7.9 (0.7)		3.3 (1.2)		< 0.001
No	184 (96.3)	7.3 (1.0)		3.7 (1.7)		< 0.001
Injection			0.816		0.492	
Yes	2 (1)	7.5 (0.7)		4.5 (2.1)		0.374
No	190 (99)	7.3 (1.0)		3.7 (1.7)		< 0.001
Water treatment			0.301		0.873	
Yes	13 (6.8)	7.6 (0.9)		3.6 (1.7)		< 0.001
No	179 (93.2)	7.3 (1.0)		3.7 (1.6)		< 0.001
Message therapy			0.684		0.440	
Yes	6 (96.9)	7.2 (1.2)		3.2 (1.5)		0.007
No	186 (3.1)	7.3 (1.0)		3.7 (1.7)		< 0.001
Yoga			0.102		0.561	
Yes	2 (1)	8.5 (0.7)		3.0 (0)		0.058
No	190 (99)	7.3 (1.0)		3.7 (16)		< 0.001
Surgery			0.254		0.308	
Yes	3 (1.6)	8.0 (0)		4.7 (3.0)		0.199
No	190 (98.4)	7.3 (1.0)		3.7 (1.6)		< 0.001

*Note:* Short duration: Up to 24 months, Long duration: 3 to 6 years; NRS: Numeric Rating Scale for pain (0–10). Acupuncture, Injection, Water treatment, Message therapy, Yoga, and Surgery not been categorized to Short & long treatment and compared due to the low number of participants.

In the adjusted general linear model, associations with post‐treatment pain intensity varied across treatment modalities. Home exercise remained independently associated with lower post‐treatment pain intensity after adjustment (regression coefficient (*β*) = −0.74, 95% CI: −1.30 to −0.18, *p* = 0.010. A similar direction of association was observed in the short‐duration subgroup, although the association was not statistically significant (*β* = −0.72, *p* = 0.09). Short‐duration pharmacological therapy (≤ 24 months) was independently associated with lower post‐treatment pain intensity (*β* = −0.96, 95% CI: −1.73 to −0.18, *p* =;0.02), while long‐term use was not significantly associated with post‐treatment pain intensity (*β* = 0.33, *p* = 0.39). Physiotherapy showed a non‐significant trend toward lower pain intensity in total (*β* = −0.51, *p* = 0.08) and long‐duration cohorts (*β* = −0.66, *p* =;0.10). Acupuncture (*β* = −0.45, *p* = 0.50) and water therapy (*β* = −0.23, *p* = 0.65) showed no significant effects (Table 4).

**Table 4:** The results of the adjusted general linear model to predict NRS score by different treatments totally and stratified by duration of treatment.

Different treatments	Adjusted *β* 95% confidence interval	*p* value€	Adjusted *β* 95% confidence interval	*p* value	Adjusted *β* 95% confidence interval	*p* value
	Total		Short duration		Long duration	
Pharmacological therapy	−0.18 (−0.72 to 0.36)	0.503	−0.96 (−1.73 to −0.18)	**0.017**	0.33 (−0.43 to 1.09)	0.389
Physiotherapy	−0.51 (−1.07 to 0.06)	0.077	−0.51 (−1.36 to 0.35)	0.241	−0.66 (−1.44 to 0.12)	0.097
Acupuncture	−0.45 (−1.75 to 0.86)	0.500	—		—	—
Water treatment	−0.23 (−1.25 to 0.78)	0.650	−0.54 (−1.88 to 0.79)	0.424	0.09 (−1.45 to 1.64)	0.900
Home exercise	−0.74 (−1.30 to −0.184)	**0.010**	−0.72 (−1.55 to 0.11)	0.086	−0.55 (−1.46 to 0.36)	0.235
Message therapy	0.24 (−1.47 to 1.95)	0.781	—	—	—	—

*Note:* € Linear regression test adjusted for BMI, occupation, gender, education level, and other received treatments, Short duration: up to 24 months, Long duration: 3 to 6 years; NRS: Numeric Rating Scale for pain. Bold values indicate significant differences.

Multivariable logistic regression models were adjusted for BMI, gender, comorbidities, and treatment modalities. In the overall cohort, participants receiving water therapy had higher odds of paresthesia (adjusted odds ratio (OR) = 4.14, 95% CI: 1.19–14.43, *p* = 0.03).

This finding persisted in the backward logistic regression (LR) model, where water treatment remained independently associated with higher odds of paresthesia (OR = 3.52, 95% CI: 1.09–11.37, *p* = 0.04). BMI also emerged as a significant factor, with normal BMI (vs. obesity) conferring a 76% reduction in paresthesia odds (OR = 0.24, 95% CI: 0.07–0.80, *p* = 0.02). No significant associations were observed for pharmacological therapy (total cohort: OR = 0.79, 95% CI: 0.37–1.69, *p* = 0.56), physiotherapy (total cohort: OR = 1.72, 95% CI: 0.77–3.81, *p* = 0.18), or home exercise (total cohort: OR = 0.89, 95% CI: 0.41–1.94, *p* = 0.78). However, short‐duration pharmacological therapy showed a borderline trend toward reduced risk (OR = 0.40, 95% CI: 0.14–1.14, *p* = 0.09), while long‐duration use suggested non‐significant higher odds (OR = 1.73, 95% CI: 0.57–5.31, *p* = 0.33) rather than other treatments. Duration‐stratified analyses revealed no statistically significant associations in the long‐duration subgroup (3–6 years) for any intervention. Water treatment data were unavailable for this subgroup for low cases (< 5). In the short‐duration subgroup (≤ 24 months), physiotherapy (OR = 2.08, 95% CI: 0.61–7.06, *p* = 0.24) and home exercise (OR = 1.19, 95% CI: 0.42–3.43, *p* = 0.74) similarly lacked significance. The Hosmer‐Lemeshow test demonstrated adequate model fit for the total cohort (*χ* ^2^ = 10.28, df = 8, *p* = 0.25), short‐duration subgroup (≤ 24 months: *χ* ^2^ = 3.27, df = 8, *p* = 0.92), and long‐duration subgroup (3–6 years: *χ* ^2^ = 8.6, df = 8, *p* = 0.38) (Table 5).

**Table 5:** The results of the multivariate logistic regression model to predict the odds ratio of paresthesia by different received treatments adjusting for confounders.

	Total		Short duration		Long duration	
Different treatments	Adjusted OR (95% CI)	*p* value^€^	Adjusted OR (95% CI)	*p* value^€^	Adjusted OR (95% CI)	*p* value^€^
Pharmacological therapy	0.79 (0.37–1.69)	0.556	0.40 (0.14–1.14)	0.087	1.73 (0.57–5.31)	0.334
Physiotherapy	1.72 (0.77–3.81)	0.181	2.08 (0.61–7.06)	0.238	1.06 (0.31–3.61)	0.924
Water treatment	4.14 (1.190–14.43)	**0.026**	—	—	—	**—**
Home exercise	0.89 (0.41 to 1.94)	0.783	1.19 (0.42 to 3.43)	0.737	0.51 (0.15 to 1.70)	0.277

*Note:* € Logistic regression with Enter model adjusted for BMI, gender, comorbidities, and treatments. Hosmer & Lemeshow total: Chi^2^: 10.28, df: 8, *p* = 0.246, Hosmer & Lemeshow short duration Chi^2^: 3.27, df: 8, *p* = 0.916, Hosmer & Lemeshow long duration: Chi^2^: 8.6, df: 8, *p* = 0.376; Short duration: up to 24 months, Long duration: 3 to 6 years. Moreover, we ran logistic regression with a backward LR model. BMI and Water treatment remained in the final step. Those with normal BMI versus obese ones (OR: 95% CI: 0.24: 0.07–0.80, *p* = 0.021) had significantly lower odds and those who received water treatment (OR: 95% CI: 3.52: 1.09–11.37, *p* = 0.036) had significantly more probability for paresthesia. Bold valuse indicate significant differences.

Satisfaction tended to be higher among participants receiving longer treatment duration, although the difference did not reach conventional statistical significance (*p* = 0.05). The majority of participants in both cohorts reported satisfaction (≥ 66%), with minimal dissatisfaction (< 5%). All treatments except injection had ≥ 75% of participants report being either “completely satisfied” or “satisfied”. The greatest proportion of dissatisfaction (33.3%) was related to home exercise. Caution is warranted in interpreting results for therapies with very small sample sizes of yoga and injection (Table 6). Complete outcome data were available for all 194 participants.

**Table 6:** The reported satisfaction rate based on different received treatments and treatment duration.

Variable	Completely satisfied	Satisfied	Not satisfied nor dissatisfied	Dissatisfied	Completely dissatisfied
Type of treatment	*n* (%)	*n* (%)	*n* (%)	*n* (%)	*n* (%)
Pharmacological therapy	24 (31.2)	34 (44.2)	17 (22.1)	2 (2.6)	0
Physiotherapy	35 (32.1)	51 (46.8)	20 (18.3)	3 (2.8)	0
Acupuncture	3 (42.9)	3 (42.9)	0	1 (14.3)	0
Water treatment	5 (38.5)	6 (46.2)	2 (15.4)	0	0
Home exercise	36 (38.3)	37 (39.4)	17 (18.1)	3 (32.2)	1 (1.1)
Message therapy	3 (50)	2 (33.3)	1 (16.7)	0	0
Injection	0	1 (50)	1 (50)	0	0
Yoga	1 (50)	1 (50)	0	0	0
Treatment duration					
6 months to 2 years (*n* = 92)	21 (22.8)	40 (43.5)	28 (30.4)	2 (2.2)	1 (1.1)
3 years to 6 years (*n* = 83)	30 (36.1)	33 (39.8)	17 (20.5)	3 (3.6)	0
Total (*n* = 175)	51 (25.6)	73 (36.7)	45 (22.6)	5 (2.5)	1 (0.5)

*Note:* Each person may have received more than one treatment during their treatment period.

## 4 Discussion

The current study revealed that BMI and Education as important factors influencing pain, as well as BMI, gender, and comorbidities as critical factors influencing paresthesia in LDH. Substantial reductions in both pain intensity and paresthesia following the treatment period suggest favorable clinical outcomes following conservative therapies in managing LDH‐related symptoms. The mean reduction of 3.6 NRS points exceeds the commonly accepted minimal clinically important difference for low back pain. The findings suggest that home exercise and short‐term pharmacological therapy were associated with more favorable pain outcomes in this cohort. However, causal inferences cannot be drawn because of the retrospective observational design. The adjusted linear regression model highlighted home exercise and short‐term pharmacological therapy as key modifiable factors for pain management. While pain intensity and paresthesia cases significantly reduced following all treatments, according to adjusted logistic regression, water therapy compared to other treatments with adequate cases and obesity (via BMI) were robust paresthesia predictors, while other interventions lacked statistical significance. Duration‐specific analyses highlighted nuanced trends but no conclusive associations. The majority of participants in both short‐term and long‐term treatments reported satisfaction (≥ 66%), with minimal dissatisfaction (< 5%). All treatments except injection had ≥ 75% “completely satisfied” or “satisfied” rate.

Our study demonstrated that BMI and education level are critical factors influencing both the prevalence and severity of pain. Supporting this finding, Su et al. (2021) investigated associations between BMI and low back pain (LBP) using data from 4796 participants. The study revealed a stepwise increase in LBP prevalence with higher BMI categories: 47.4% of normal/underweight individuals reported LBP versus 72.8% of morbidly obese patients. Moreover, similar studies consistently show obese patients reported higher pain intensity and interference compared to normal‐weight individuals, and obesity was identified as an important independent predictor of back pain and its severity [26, 27]. The relationship between obesity and pain is likely multifaceted, involving mechanical stress, metabolic disruptions, and lifestyle factors [28]. The accumulation of adipose tissue in obesity leads to increased production of pro‐inflammatory cytokines and decreased anti‐inflammatory cytokines, exacerbating inflammation and pain [29]. Physical activity has been proposed as an intervention to reduce systemic inflammation and alleviate pain in obese individuals, though adherence to exercise programs remains a challenge [30]. Moreover, consistent with our result, extensive research confirms sex‐specific variations in pain processing and therapeutic responses, with women exhibiting higher clinical pain prevalence, greater pain‐related psychological distress, and enhanced sensitivity to experimentally induced stimuli compared to men [31]. Female patients with LDH exhibit lower heat and pressure pain thresholds than males [32]. In chronic non‐cancer pain conditions, females report higher baseline pain intensity and greater use of certain medications, while males consume more opioids [33]. These differences may be attributed to various factors, including psychosocial elements like pain‐related catastrophizing and biological processes such as gonadal hormone levels [31].

The relationship between paresthesia and obesity may involve mechanical pathways. Obesity increases mechanical stress on the lumbar spine, contributing to disc degeneration, herniation, and sciatica. Zhou et al.'s randomization study revealed obesity causally elevates dorsopathy risks, including sciatica, low back pain, and disc degeneration, with mechanical stress from fat mass/hip circumference and sedentary behavior (mediating 30%–50% effects) contributing to root compression and paresthesia. Fat‐free mass independently increased disc degeneration risk, suggesting obesity‐driven spinal strain may exacerbate nerve compression [34]. Increased BMI is associated with greater disc deformation, particularly at the L5–S1 level, following physical activity [35]. Furthermore, a cross‐sectional study of 2599 adults revealed a significant positive correlation between BMI and the presence, extent, and severity of lumbar disc degeneration on MRI, with obese individuals having 1.79 times higher odds of disc degeneration compared to normal‐weight individuals [36]. Abdominal adiposity also affects spinal curvature, particularly increasing lumbar lordosis [37]. This change in spinal alignment, along with abdominal obesity, doubles the risk of low back pain [37]. These findings highlight the importance of weight management in preventing and managing lumbar spine disorders.

Research indicates a significant relationship between education and pain perception. Lower educational attainment is associated with higher postoperative pain levels and increased pain prevalence in older adults [38, 39]. Among adults aged 30–49, a steep pain gradient exists, with higher education generally linked to less pain [40]. Zajacova et al. [41] reveal a significant inverse correlation between educational attainment and pain prevalence among U.S. adults aged 30–49, where higher education correlates with reduced pain. Notably, adults with a high‐school equivalency diploma or partial college education report greater pain levels than high school graduates, while Hispanic populations exhibit no such educational gradient. With over half of this demographic experiencing pain, the study emphasizes the need to investigate social determinants such as systemic stressors or inequities that drive elevated pain in specific educational subgroups, challenging conventional assumptions about socioeconomic status and health outcomes. Pain Neuroscience Education (PNE) programs have shown potential in reducing pain perception among elderly individuals with chronic pain [41]. These findings highlight the importance of considering educational background in pain management strategies.

Our research demonstrated that comorbidities, particularly hypertension and gastrointestinal disorders, showed significant associations with paresthesia. These conditions may contribute to paresthesia development through multiple pathophysiological pathways. Gastrointestinal disorders can significantly impair nutrient absorption, leading to various micronutrient deficiencies. Micronutrient deficiencies can lead to various neurological symptoms, including paresthesia. Vitamin B12 deficiency, in particular, can cause both central and peripheral paresthesia, which is partially reversible with treatment [42]. On the other hand, spinal cord stimulation for chronic neuropathic pain can sometimes lead to gastrointestinal side effects, possibly due to interference with parasympathetic outflow [43]. These findings highlight the complex relationship between spinal conditions and gastrointestinal function, emphasizing the importance of considering spinal pathologies in patients presenting with unexplained gastrointestinal symptoms.

Hypertension has been independently associated with moderate to severe lumbar disc degeneration, increasing the likelihood by 50% [44]. Hypertension is also associated with structural changes in the vascular supply to peripheral nerves, leading to potential ischemia and impaired nerve function [45]. This phenomenon might be attributed to the fact that NSAIDs are commonly used to manage pain in LDH patients by reducing inflammation and relieving mild‐to‐moderate discomfort; however, their use requires caution in specific scenarios [16]. NSAIDs (e.g., indomethacin, diclofenac, and naproxen) may elevate blood pressure, potentially worsening vascular dysfunction and reducing nerve perfusion, which could indirectly exacerbate neuropathy symptoms like paresthesia [46, 47, 48].

Our analysis identified an association between water therapy and higher odds of paresthesia; however, this finding should be interpreted as hypothesis‐generating rather than clinically actionable. The small number of participants receiving water therapy may have resulted in unstable estimates and susceptibility to sparse‐data bias. Further prospective studies with larger samples are required to clarify this observation. In related research, hydrotherapy has shown promise in managing pain and improving functional ability in patients with LDH [49]. Pain reduction following water therapy may paradoxically increase awareness of paresthesia. This might occur because as pain diminishes, residual nerve compression or irritation could persist, causing pre‐existing paresthesia to become more apparent once acute pain subsides.

In the current study, substantial reductions in pain intensity and paresthesia were observed following conservative treatment. Although these findings are encouraging, improvements cannot be attributed solely to the interventions because of the absence of an untreated comparison group and the observational study design. Badr et al. [50] demonstrated that a multimodal physical therapy approach has been found to significantly improve pain, disability, and nerve root parameters in chronic LDH patients. Exercise and traction are also strongly recommended, while epidural injections, particularly the transforaminal approach, have shown high efficacy [51].

Our findings highlight home exercise and short‐term pharmacological therapy as particularly effective strategies, while underscoring the need for larger trials on niche interventions. Extensive research, including randomized controlled trials and systematic reviews, corroborates the efficacy of home‐based exercise regimens in improving functional outcomes and reducing pain intensity among patients with LDH. For instance, Arslan's systematic review of 12 RCTs (*n* = 880) found home‐based exercise interventions significantly improved outcomes in LDH. These exercises reduced pain, enhanced functional capacity and quality of life, and improved transversus abdominis activation compared to other approaches. Results align with evidence supporting structured home exercise as a key conservative therapy for LDH management [52]. A randomized trial in 59 first‐time lumbar disc surgery patients revealed that structured home‐based exercise programs achieved significantly greater pain reduction (*p* < 0.05) and quality‐of‐life improvements compared to clinic‐based physiotherapy at 3–12 month follow‐ups. While clinic‐based care yielded higher long‐term activity levels (+15%) and short‐term satisfaction, home exercise demonstrated equal efficacy in reducing disability [53]. Aligned with our findings, a systematic review of RCTs confirmed that pharmacological therapies, particularly NSAIDs like ibuprofen, achieve short‐term reductions in acute pain and inflammation in LDH by specifically addressing nerve root irritation mechanisms. However, as previously mentioned, long‐term reliance on NSAIDs or steroids is limited by diminishing efficacy and risks of gastrointestinal, cardiovascular, and renal complications [54, 55]. While drugs offer temporary relief, studies emphasize combining pharmacological therapy with physical therapy or lifestyle modifications to address structural causes and prevent recurrence [12, 18].

This study has several limitations inherent to its retrospective observational design. First, the reliance on medical records and patient recall introduces potential selection bias and information bias, particularly regarding self‐reported pain intensity and treatment adherence. The single‐center design and recruitment from specialized rehabilitation clinics may limit generalizability to broader populations or different healthcare settings. Second, while the sample size was adequate for primary outcomes, subgroup analyses (particularly for less common interventions like acupuncture, yoga, and surgery) were underpowered due to small sample sizes, potentially obscuring true treatment effects. Another limitation was the heterogeneity of conservative treatment regimens. Many patients received combinations of interventions, and detailed information regarding the exact sequence, intensity, and overlap of treatment modalities was not consistently available from retrospective records. Therefore, regression estimates should be interpreted cautiously. Furthermore, while the study effectively identifies causal relationships, its precision is limited due to the observational nature of the study. Additionally, some outcome information, including subsequent surgery, was obtained retrospectively. The exact timing and clinical decision‐making leading to surgery may not have been fully captured, which limits interpretation of surgery as a definitive marker of conservative treatment failure. Finally, lack of detailed information regarding treatment dose, intensity, frequency, and procedural characteristics limited assessment of treatment reproducibility. To overcome these constraints, future investigations should employ prospective, multi‐center methodologies utilizing adequately powered sample sizes that incorporate heterogeneous patient cohorts across varied clinical contexts, thereby optimizing the external validity and translational applicability of research outcomes.

## 5 Conclusion

This study suggests that certain conservative treatment modalities, particularly home exercise and short‐term pharmacological therapy, were associated with improved pain outcomes among patients with LDH. However, prospective controlled studies are required to determine comparative effectiveness and causal relationships. Key modifiable factors like BMI, education, and comorbidities highlight the value of personalized, non‐invasive strategies and integration of lifestyle modifications. Despite robust short‐term efficacy, long‐term evaluation of conservative regimens remains critical to assess durability, recurrence rates, and cost‐effectiveness compared to other modalities such as surgery. Future approaches to treating LDH should focus on comprehensive, patient‐centered care, relying on proven conservative therapies.

## Author Contributions

**Bina Eftekharsadat:** conceptualization, investigation, writing – original draft, writing – review and editing, conceptualization. **Azizeh Farshbaf‐Khalili:** data curation, formal analysis, methodology, investigation, writing – original draft. **Soraya Babaie:** conceptualization, investigation, writing – review and editing. **Shadi Khodaei, Raha Markazi‐Movaghar**, and **Fatemeh Ojaghlou:** investigation, data curation, writing – review and editing. All authors have read and approved the final version of the submitted manuscript and agreed to be accountable for all aspects of the work. The corresponding author had full access to all of the data in this study and takes complete responsibility for the integrity of the data and the accuracy of the data analysis.

## Ethics Statement

The study was approved by the Ethics Committee of Tabriz University of Medical Sciences on April 10, 2025 (approval code: IR.TBZMED.REC.1403.1112) and authorization from the Vice Chancellor of Research.

## Conflicts of Interest

The authors declare no conflicts of interest.

## Transparency Statement

Dr. Soraya Babaie affirms that this manuscript is an honest, accurate, and transparent account of the study being reported; that no important aspects of the study have been omitted.

## Data Availability

The data that support the findings of this study are available from the corresponding author upon reasonable request. All data generated or analyzed during this study are included in this published article.

## Appendix 1

### 1.1

Table A1.

**Table A1:** The number of the LDH patients with paresthesia after treatment (*n* = 45) stratified by received treatments.

Variable	Paresthesia after treatment (*n* = 45)
Pharmacological therapy/total	***n* (%)**
Yes	19 (42.2%)
No	26 (57.8%)
Physiotherapy	
Yes	31 (68.9%)
No	14 (31.1%)
Acupuncture	
Yes	2 (4.5%)
No	43 (95.5%)
Injection	
Yes	1 (2.2%)
No	44 (97.8%)
Water treatment	
Yes	6 (1.3%)
No	39 (86.7%)
Home exercise	
Yes	22 (48.9%)
No	23 (51.1%)
Message therapy	
Yes	1 (2.2%)
No	44 (97.8%)
Yoga	
Yes	1 (2.2%)
No	44 (97.8%)
Surgery	
Yes	3 (6.5%)
No	43 (93.5%)
